# Predictors of deep-vein thrombosis for acute stroke at admission to a rehabilitation unit: A retrospective study

**DOI:** 10.3389/fneur.2023.1137485

**Published:** 2023-03-31

**Authors:** Fang Li, Changkun Wei, Su Huo, Xiuzhen Liu, Jubao Du

**Affiliations:** ^1^Department of Rehabilitation Medicine, Xuanwu Hospital, Capital Medical University, Beijing, China; ^2^School of Mathematics and Statistics, Beijing Jiaotong University, Beijing, China

**Keywords:** deep-vein thrombosis (DVT), acute stroke, Berg Balance Scale (BBS), D-dimer, rehabilitation

## Abstract

**Background:**

Deep-vein thrombosis (DVT) is a common complication of acute stroke (AS). Only limited studies have discussed DVT in patients with AS at admission to a rehabilitation unit. The purpose of this study is to identify the predictors of DVT in AS patients admitted to a rehabilitation unit in China.

**Methods:**

We retrospectively reviewed the medical records of all patients with AS admitted within 14 days of stroke onset between July 2019 and June 2022 at the Department of Rehabilitation Medicine, Xuanwu Hospital, Capital Medical University, China. Ultrasonography was used to diagnose DVT in all patients within 3 days after rehabilitation admission. Univariate and binary logistic regression analyses were performed to determine the risk factors for DVT.

**Results:**

Overall, 234 cases were identified and the incidence rate of DVT among AS patients was 13.2% (31/234). The univariate analysis showed that age, drinking, lower limb muscle strength, Brunnstrom Assessment (BRS), Fugl-Meyer Assessment (FMA), Berg Balance Scale (BBS), Barthel Index (BI) scale, serum albumin (Alb), and D-dimer were statistically significant factors. Age (OR = 1.037, 95% CI = 1.000–1.075, *p* < 0.05), BBS (OR = 0.952, 95% CI = 0.913–0.993, *p* < 0.05), and D-dimer (OR = 1.446, 95% CI = 1.130–1.849, *p* < 0.05) were demonstrated as independent risk factors for DVT.

**Conclusion:**

Older age, lower BBS, and higher D-dimer levels at rehabilitation admission were independent risk factors for DVT. Therefore, ultrasonography should be performed for those patients with these three significant factors before implementing rehabilitation therapy.

## 1. Introduction

Acute stroke (AS) is a major cause of neurological disability and mortality among hospitalized patients (1). A considerable proportion of survivors demonstrate paralysis for a period after the stroke. These stroke survivors often require early rehabilitation services after their condition stabilizes as rehabilitation services are the primary mechanism by which functional recovery and the achievement of independence are promoted in patients with AS (2). However, paralysis after AS can cause various complications, such as deep-vein thrombosis (DVT), amyotrophy, or pressure ulcers (3). Among them, DVT is one of the most common and fatal complications for patients with AS and is one of the leading causes of hindering the early rehabilitation process (4, 5).

Deep-vein thrombosis is mainly caused by abnormal blood coagulation in the deep veins and venous reflux disease, which often affects the lower limbs and can result in pulmonary embolism (PE) (6–8). Reduced mobility is an important risk factor for DVT; thus, AS patients are considered to have a high risk of developing DVT (9). The incidence rate of DVT among post-stroke patients ranges from 10 to 80%, depending on the diagnostic approach, time of evaluation, and pharmacological thromboprophylaxis (6, 10–12). Moreover, symptomatic DVT and asymptomatic DVT after AS vary in their prevalence, with the former being 2–20% (10, 11, 13). Furthermore, DVT development may occur as early as the 2nd day, peaking between 2 and 7 days (9, 11). Since the existence of DVT can lead to delaying rehabilitation intervention, the risk of developing DVT in patients with AS should be evaluated early to actively intervene to prevent DVT. In this study, we aim to evaluate clinical and laboratory variables specific to the presence of DVT at admission to the department of rehabilitation medicine for AS patients, in order to identify patients who could benefit from a more aggressive screening strategy.

## 2. Methods

### 2.1. Participants

We retrospectively reviewed 234 patients with AS admitted to the Department of Rehabilitation Medicine, Xuanwu Hospital, Capital Medical University, from July 2019 to June 2022. The eligible patients were required to meet the following criteria: patients (1) who were aged ≥18 years; (2) whose stroke onset time <14 days at rehabilitation admission; and (3) who performed an ultrasound examination within 3 days after rehabilitation admission. The exclusion criteria were as follows: patients with (1) a previous medical history of AS; (2) a history of venous thromboembolism (VTE); (3) varicose of lower extremities; (4) a history of lower limb surgery; and (5) incomplete medical records.

### 2.2. Diagnosis of DVT

Deep-vein thrombosis was diagnosed according to the findings of lower extremity ultrasonography, performed by trained ultrasound physicians. DVT was defined based on the following ultrasonic findings: the presence of a non-compressible segment or flow impairment on color Doppler imaging (14). Compression was performed at 2-cm intervals (15).

### 2.3. Data collection

Based on electronic medical records, the demographic information of patients was collected at rehabilitation admission, including age, gender, body mass index (BMI), smoking and drinking, history of surgery (excluding lower limb surgery), history of thrombolysis, comorbidities (hypertension, hyperlipidemia, diabetes, coronary heart disease, atrial fibrillation, and malignant tumor), and infections (pneumonia and urinary tract infection). The clinical variables within 24 h at hospitalization were collected, including the onset-to-rehabilitation admission time, site of stroke (cerebral hemisphere, brainstem, and both), lower extremity manual muscle testing (MMT) and muscle tone, Brunnstrom Assessment (BRS), Fugl-Meyer Assessment (FMA), Berg Balance Scale (BBS), and Barthel index (BI) scale. The following laboratory data were obtained with blood samples collected within 24 h after hospitalization: white blood cell (WBC) count, hematocrit, red blood cell (RBC) count, red blood cell volume distribution width (RDW), hemoglobin (Hb), platelet (PLT) count, serum albumin (Alb), triglycerides (TG), total cholesterol, high-density lipoprotein cholesterol (HDL-C), low-density lipoprotein cholesterol (LDL-C), glucose, serum uric acid (SUA), aspartate aminotransferase (AST), alanine aminotransferase (ALT), fibrinogen, D-dimer, activated partial thromboplastin time (APTT), and prothrombin time (PT).

### 2.4. Statistics

The data were gathered in a Microsoft Excel spreadsheet by two researchers and then cross-checked two times to assure information accuracy. Statistical analysis was performed using IBM SPSS version 26.0. Continuous variables were analyzed in the form of the means with standard deviations (Mean ± SD). Categorical variables were shown as numbers and proportions. Continuous data were analyzed using the independent samples *t*-test and the Mann–Whitney *U*-test. Categorical data were analyzed using the chi-squared test or Fisher's exact test, as appropriate. Univariate variables with a *p*-value of ≤0.1 were retained in a binary logistic regression model. Backward elimination was employed to select the final predictors that were independently associated with DVT. Receiver operating characteristic (ROC) curve analysis was performed by identifying the area under the ROC curve (AUC) to evaluate the performance of the logistic regression model. Statistical significance was defined as a *p*-value of < 0.05.

## 3. Results

### 3.1. General characteristics of the patients

A total of 234 patients with AS were included in the study. The included patients were divided into two groups: DVT (*n* = 31) and non-DVT group (*n* = 203). The general characteristics of the patients are demonstrated in Table 1. There was no significant difference in gender, BMI, smoking, history of surgery, history of thrombolysis, comorbidities, and infections between the two groups. Patients with DVT were older than those without DVT (64.7 ± 11.7 vs. 59.5 ± 11.9 years, *p* < 0.05). Drinking was overrepresented in patients without DVT (*p* < 0.05).

**Table 1:** Demographic details of the study population at rehabilitation admission.

**Category**	**Total (*N* = 234)**	**DVT (*N* = 31)**	**Non-DVT (*N* = 203)**	***P*** **-value**
**Gender**				0.793
Male, *n* (%)	178 (76.1)	23 (74.2)	155 (76.4)	
Female, *n* (%)	56 (23.9)	8 (25.8)	48 (23.6)	
Age (years)	60.2 ± 12.0	64.7 ± 11.7	59.5 ± 11.9	0.034
BMI (kg/m^2^)	25.9 ± 3.6	25.8 ± 3.1	26.0 ± 3.6	0.963
Smoking, *n* (%)	118 (50.4)	11 (35.5)	107 (52.7)	0.074
Drinking, *n* (%)	124 (53.0)	11 (35.5)	113 (55.7)	0.036
History of surgery, *n* (%)	95 (40.6)	10 (32.3)	85 (41.9)	0.310
History of thrombolysis, *n* (%)	43 (18.4)	8 (25.8)	35 (17.2)	0.251
**Comorbidities**				
Hypertension, *n* (%)	176 (75.2)	24 (77.4)	152 (74.9)	0.760
Hyperlipidemia, *n* (%)	82 (35.0)	13 (41.9)	69 (44.0)	0.388
Diabetes, *n* (%)	88 (37.6)	11 (35.5)	77 (37.9)	0.793
Coronary heart disease, *n* (%)	27 (11.5)	4 (12.9)	23 (11.3)	0.765
Atrial fibrillation, *n* (%)	10 (4.3)	2 (6.5)	8 (3.9)	0.626
Malignant tumor, *n* (%)	4 (1.7)	1 (3.2)	3 (1.5)	0.436
**Infections**				
Pneumonia, *n* (%)	26 (11.1)	1 (3.2)	25 (12.3)	0.217
Urinary tract infection, *n* (%)	7 (3.0)	2 (6.5)	5 (2.5)	0.234

DVT, deep-vein thrombosis; BMI, body mass index.

Table 2 shows the details of clinical variables in those with and without DVT. Participants with DVT had worse lower limb muscle strength as indicated by MMT (1.6 ± 1.5 vs. 2.3 ± 1.6, *p* < 0.05). There was a significantly worse balance function in the DVT group (5.1 ± 10.2 vs. 16.7 ± 17.7, *p* < 0.001), as represented by a lower BBS on admission. Patients in the DVT group had more severe motor dysfunction as indicated by BRS and FMA (2.7 ± 1.2 vs. 3.4 ± 1.3, *p* < 0.05; 34.7 ± 21.0 vs. 48.5 ± 27.7, *p* < 0.05). Compared to the non-DVT group, patients in the DVT group had significant functional inability, with lower BI scores (37.3 ± 17.2 vs. 48.8 ± 21.8, *p* < 0.05). No other clinical factors for AS were associated with DVT.

**Table 2:** Clinical characteristics of the patients at rehabilitation admission.

**Variables**	**Total (*N* = 234)**	**DVT (*N* = 31)**	**Non-DVT (*N* = 203)**	***P*** **-value**
Onset-to-rehabilitation admission time (days)	10.5 ± 2.4	10.3 ± 2.3	10.5 ± 2.4	0.677
**Site of stroke**				1.000
Cerebral hemisphere, *n* (%)	179 (76.5)	24 (77.4)	155 (76.4)	
Brainstem, *n* (%)	52 (22.2)	7 (22.6)	45 (22.2)	
Both, *n* (%)	3 (1.3)	0 (0.0)	3 (1.5)	
Lower extremity MMT (grade)	2.2 ± 1.6	1.6 ± 1.5	2.3 ± 1.6	0.017
**Lower extremity**				0.144
**muscle tone**				
Increased, *n* (%)	6 (2.6)	1 (3.2)	5 (2.5)	
Normal, *n* (%)	204 (87.2)	24 (77.4)	180 (88.7)	
Decreased, *n* (%)	24 (10.3)	6 (19.4)	18 (8.9)	
BRS (grade)	3.3 ± 1.3	2.7 ± 1.2	3.4 ± 1.3	0.007
FMA	46.7 ± 27.3	34.7 ± 21.0	48.5 ± 27.7	0.008
BBS	15.1 ± 17.4	5.1 ± 10.2	16.7 ± 17.7	<0.001
BI	47.3 ± 21.6	37.3 ± 17.2	48.8 ± 21.8	0.005

DVT, deep-vein thrombosis; BRS, Brunnstrom Assessment; FMA, Fugl-Meyer Assessment; BBS, Berg Balance Scale; BI, Barthel index.

Table 3 shows the results of laboratory factors in patients with DVT vs. those without DVT. Lab variables (WBC, hematocrit, RBC, RDW, Hb, PLT, TG, total cholesterol, HDL-C, LDL-C, glucose, SUA, AST, ALT, fibrinogen, APTT, and PT) were not significantly different between the two groups. In the DVT group, the level of D-dimer was significantly higher than that in the non-DVT group (1.9 ± 1.7 vs. 0.7 ± 1.1, *p* < 0.001), while the level of Alb was significantly lower in the DVT group than the non-DVT group (36.3 ± 3.9 vs. 38.5 ± 3.5, *p* = 0.001).

**Table 3:** Laboratory predictors of DVT at rehabilitation admission.

**Parameters**	**Total (*N* = 234)**	**DVT (*N* = 31)**	**Non-DVT (*N* = 203)**	***P*** **-value**
WBC (10^∧^9/L)	7.7 ± 2.0	7.6 ± 1.6	7.7 ± 2.0	0.871
Hematocrit (%)	42.5 ± 4.7	41.7 ± 3.9	42.6 ± 4.8	0.153
RBC (10^∧^12/L)	4.7 ± 0.6	4.5 ± 0.5	4.7 ± 0.6	0.165
RDW (%)	12.7 ± 1.2	12.7 ± 0.5	12.7 ± 1.2	0.286
Hb (g/L)	143.2 ± 17.0	140.6 ± 15.4	143.6 ± 17.2	0.190
PLT (10^∧^9/L)	254.4 ± 77.3	239.2 ± 84.0	256.7 ± 76.2	0.092
Alb (g/L)	38.2 ± 3.6	36.3 ± 3.9	38.5 ± 3.5	0.001
TG (mmol/L)	1.4 ± 0.6	1.4 ± 0.7	1.4 ± 0.6	0.614
Total cholesterol (mmol/L)	3.2 ± 0.7	3.3 ± 0.8	3.2 ± 0.7	0.393
HDL-C (mmol/L)	0.9 ± 0.2	0.9 ± 0.2	0.9 ± 0.2	0.590
LDL-C (mmol/L)	1.7 ± 0.6	1.9 ± 0.6	1.7 ± 0.6	0.329
Glucose (mmol/L)	6.3 ± 2.3	6.1 ± 1.9	6.4 ± 2.4	0.729
SUA (umol/L)	283.5 ± 94.7	259.7 ± 68.1	287.2 ± 97.7	0.055
AST (IU/L)	30.1 ± 13.2	29.8 ± 14.1	30.2 ± 13.1	0.960
ALT (IU/L)	35.4 ± 27.5	34.2 ± 30.8	35.6 ± 27.0	0.460
Fibrinogen (g/L)	4.1 ± 1.3	4.3 ± 1.2	4.0 ± 1.3	0.119
D-dimer (ug/mL)	0.8 ± 1.3	1.9 ± 1.7	0.7 ± 1.1	< 0.001
APTT (s)	36.7 ± 4.6	37.6 ± 4.3	36.6 ± 4.6	0.158
PT (s)	12.9 ± 0.7	12.8 ± 0.7	12.9 ± 0.7	0.783

DVT, deep-vein thrombosis; WBC, white blood cell; RBC, red blood cell; RDW, red blood cell volume distribution width; Hb, hemoglobin; PLT, platelet; Alb, serum albumin; TG, triglycerides; HDL-C, high-density lipoprotein cholesterol; LDL-C, low-density lipoprotein cholesterol; SUA, serum uric acid; AST, aspartate aminotransferase; ALT, alanine aminotransferase; APTT, activated partial thromboplastin time; PT, prothrombin time.

### 3.2. Binary logistic regression analysis of variables correlated to DVT

In total, three independent variables were identified by binary logistic regression analysis in Table 4.

**Table 4:** Binary logistic regression analysis of variables correlated to DVT at rehabilitation admission.

**Variables**	**B**	**OR**	**95% CI**	***P*** **-value**
Age	0.036	1.037	1.000–1.075	0.049
BBS	−0.049	0.952	0.913–0.993	0.021
D-dimer	0.368	1.446	1.130–1.849	0.003

OR, odds ratio; CI, confidence interval; BBS, Berg Balance Scale.

Younger, higher BBS, and lower D-dimer levels were associated with a lower risk of DVT [age: odds ratio (OR) = 1.037, 95% CI = 1.000–1.075, *p* < 0.05; BBS: OR = 0.952, 95% CI = 0.913–0.993, *p* < 0.05; D-dimer: OR = 1.446, 95% CI = 1.130–1.849, *p* < 0.05].

### 3.3. Prediction of DVT using ROC curves

**Figure 1:**
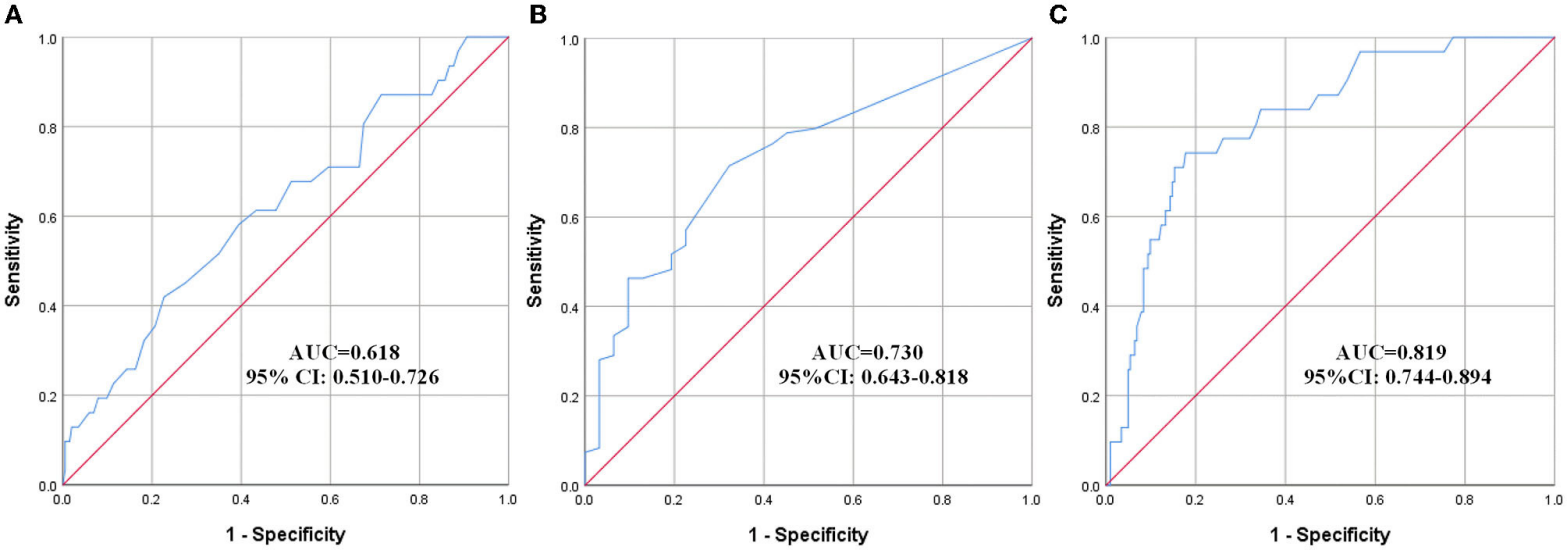
ROC curves showing a relationship between clinical, laboratory factors, and DVT. **(A)** Age, **(B)** BBS, and **(C)** D-dimer. ROC, receiver operating characteristic; AUC, area under the curve; CI, confidence interval.

**Figure 2:**
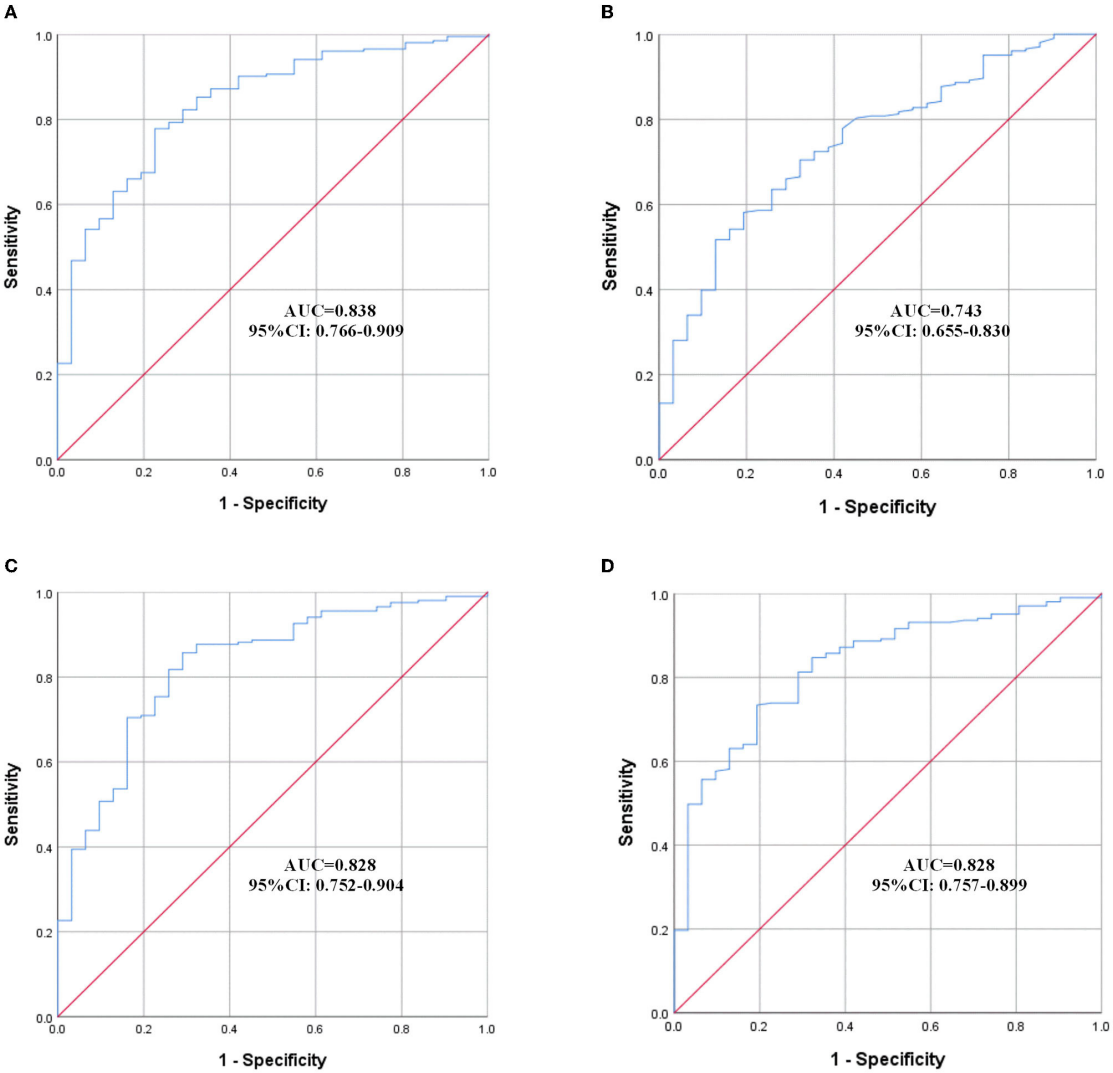
ROC curves of the combined model. **(A)** Combination of age, BBS, and D-dimer; **(B)** combination of age and BBS; **(C)** combination of age and D-dimer; **(D)** combination of BBS and D-dimer. ROC, receiver operating characteristic; AUC, area under the curve; CI, confidence interval.

The effect of variables in predicting DVT using ROC curves is shown in Table 5. Seven distinct ROC curves were illustrated: ROC curve for age (Figure 1A); ROC curve for BBS (Figure 1B); ROC curve for D-dimer (Figure 1C); combined ROC curve for age, BBS, and D-dimer (Figure 2A); combined ROC curve for age and BBS (Figure 2B); combined ROC curve for age and D-dimer (Figure 2C); and combined ROC curve for BBS and D-dimer (Figure 2D). The reported ROC curve for age had an AUC of 0.618 with the Youden index identified at 67.5 years. Similarly, the AUC of BBS and D-dimer was 0.730 and 0.819, respectively, and the Youden index value was 3.5 and 0.875 ug/ml, respectively. In addition, the combined model improved the ability of these three factors in predicting the DVT risk when compared with the three factors alone. Moreover, the AUC of the combination model in the descending order was a combination of age, BBS, and D-dimer > combination of age and D-dimer = combination of BBS and D-dimer > combination of age and BBS (Table 5 and Figure 2).

**Table 5:** Prediction of DVT at rehabilitation admission using ROC curves.

**Variables**	**Cut-off**	**Sensitivity**	**Specificity**	**AUC**	**95% CI**	***P*** **-value**
Age (years)	67.5	41.9%	79.3%	0.618	0.510–0.726	0.034
BBS	3.5	71.4%	67.7%	0.730	0.643–0.818	<0.001
D-dimer	0.875	74.2%	82.3%	0.819	0.744–0.894	<0.001
Combined (age and BBS)				0.743	0.655–0.830	<0.001
Combined (age and D-dimer)				0.828	0.752–0.904	<0.001
Combined (BBS and D-dimer)				0.828	0.757–0.899	<0.001
Combined (age, BBS, and D-dimer)				0.838	0.766–0.909	<0.001

BBS, Berg Balance Scale; AUC, area under the curve; CI, confidence interval.

## 4. Discussion

Deep-vein thrombosis was a thorny issue in rehabilitation therapy. If not properly handled, it would cause serious problems and even endanger the patients' lives. Therefore, simple and reliable measures should be taken to determine whether patients with AS had DVT before implementing rehabilitation therapy.

To the best of our knowledge, this was the first study to examine the risk factors of DVT in patients with AS at rehabilitation admission, and it was found that the incidence rate of DVT among AS populations was 13.2% (31/234), which was consistent with the occurrence of DVT after stoke varied in 3–17% in Asia (8). However, another study showed that the prevalence of DVT in stroke patients admitted to a rehabilitation unit in Singapore was 9%, which was lower than ours (9). One reason for this was that the two studies screened stroke patients with different times of onset, averaging 23.4 and 10.5 days, respectively.

Risk factors for DVT in patients after AS varied in different clinical research. The typical factors included older age, medical history of VTE, increased BMI, malignant tumor, pneumonia, and altered level of some clinical and laboratory variables (16–18). In our study, older age, lower BBS, and higher D-dimer were identified as independent factors associated with the occurrence of DVT in patients with AS at admission to a rehabilitation unit.

Previous studies have shown that advanced age was significantly associated with DVT (19, 20). Particularly, the age ≥65 years was closely related to the occurrence of DVT (6, 11). In the present study, age at a cutoff of ≥67.5 years should raise clinical suspicion and could be used as an independent factor to predict DVT risk. Because with the increase in age, blood would gradually enter a state of hypercoagulability, and mobility would also gradually decline (3, 21). Hypercoagulability and reduced mobility were easy to promote the formation of DVT, which was particularly prominent among elderly patients (22).

The BBS consisted of 14 items, scored from 0 to 4, which were added to make a total score between 0 and 56 (23). Scores of 0–20 represented balance impairment, 21–40 represented acceptable balance, and 41–56 represented good balance (24). The lower the BBS score, the worse the balance. AS patients with poor balance may be inactive or less active due to fear of falling or falling out of bed. However, immobility or restriction of mobility, a well-established risk factor, was confirmed as an important clinical tool in the assessment of patients with suspect DVT (25).

D-dimer was a breakdown product of venous thrombus and was an indicator for predicting DVT in different disorders (17, 26, 27). Patients with a higher level of D-dimer had a higher risk of DVT (28). Previous research reported that D-dimer had a sensitivity of 85–95% and a specificity of 25–50% for DVT, and a cutoff value of 0.5 μg/ml for D-dimer levels showed a sensitivity of 82.9% and a specificity of 32.7% for detecting DVT in patients during hospitalization (15, 29, 30). However, in our study, an elevated D-dimer was defined as 0.875 μg/ml; this cutoff demonstrated a sensitivity of 74.2% and a specificity of 82.3% for detecting DVT in patients with AS at rehabilitation admission. Therefore, D-dimer was a significant factor in the presence of DVT at rehabilitation admission. Given that D-dimer levels could be easily measured in any institution, D-dimer levels should be routinely examined for patients with AS at rehabilitation admission.

In addition, it was notable that our results showed that the OR of the correlation between DVT and D-dimer was 1.466 (95% CI: 1.130–1.849), while a recent meta-analysis reported a positive association between elevated D-dimer and DVT among AS patients, with an OR of 3.25 (95% CI: 2.31–4.58) (31). The difference may be attributed to the different groupings of the two studies. Our research was divided into the DVT and non-DVT groups, and a logistic regression analysis was employed to explore the correlation between D-dimer and DVT. While the meta-analysis was divided into elevated D-dimer and non-elevated D-dimer groups, observing the occurrence of VTE, inclusive of DVT, PE, or VTE-related death within 90 days, the meta-analytic approach was used to estimate the association between D-dimer and VTE.

The reported ROC curve for age, BBS, and D-dimer showed high AUC, respectively, indicating high predictive performance. Moreover, the combined ROC curve of the three variables revealed a higher AUC than the ROC curve for the two combinations of them. However, the ROC curve for age and BBS showed a lower AUC than the ROC curve for D-dimer alone. This made us believe that the presence of bivariate or trivariate variables containing D-dimer might represent a very valuable tool for DVT prediction. As a result, when patients with AS are admitted to the rehabilitation center, clinicians could quickly evaluate the abovementioned three indicators, with an emphasis on D-dimer, to determine the risk of DVT, so as to further select effective tools, such as ultrasonography, to identity whether DVT existed. The results of this study have important guiding significance, especially for those grassroots hospitals where medical conditions are insufficient to perform an ultrasound examination for every patient admitted to the rehabilitation unit.

## 5. Limitations

Our study has several limitations. First, this study was a single-center study with a small sample size, which may have led to decreased generalizability. Second, we only selected the clinical and laboratory variables on the day after rehabilitation admission and did not make any follow-up checks. Third, because all the patients were Chinese, the generalization of the study outcomes to non-Chinese populations may not be possible. Fourth, this study did not distinguish between newly developed DVT and asymptomatic DVT that had not been found prior to stroke onset during the rehabilitation hospitalization, which represented the prognostic and diagnostic value of D-dimer, respectively. A further multicenter study with larger samples was required for comprehensively analyzing the predictive value of different variables for DVT in AS patients at admission to a rehabilitation unit.

## 6. Conclusion

Despite these limitations, our study showed that older age, lower BBS, and elevated D-dimer levels were associated with the presence of DVT for patients with AS at rehabilitation admission. Therefore, ultrasonography should be performed for those patients with these three significant factors before implementing rehabilitation therapy.

## Data availability statement

The original contributions presented in the study are included in the article/supplementary material, further inquiries can be directed to the corresponding author.

## Ethics statement

Ethical review and approval was not required for the study on human participants in accordance with the local legislation and institutional requirements. Written informed consent from the patients/participants or patients/participants' legal guardian/next of kin was not required to participate in this study in accordance with the national legislation and the institutional requirements.

## Author contributions

JD and FL conceived and designed the research. CW, SH, and XL participated in analyzing the data. FL, SH, and JD wrote and revised the article. All authors read, revised, and approved the final manuscript.
